# Determining the Bending Rigidity of Free-Standing Planar Phospholipid Bilayers

**DOI:** 10.3390/membranes13020129

**Published:** 2023-01-19

**Authors:** Oscar Zabala-Ferrera, Paige Liu, Peter J. Beltramo

**Affiliations:** Department of Chemical Engineering, University of Massachusetts, Amherst, MA 01003, USA

**Keywords:** bending rigidity, planar biomembrane, lipid bilayer, asymmetric membranes

## Abstract

We describe a method to determine membrane bending rigidity from capacitance measurements on large area, free-standing, planar, biomembranes. The bending rigidity of lipid membranes is an important biological mechanical property that is commonly optically measured in vesicles, but difficult to quantify in a planar, unsupported system. To accomplish this, we simultaneously image and apply an electric potential to free-standing, millimeter area, planar lipid bilayers composed of DOPC and DOPG phospholipids to measure the membrane Young’s (elasticity) modulus. The bilayer is then modeled as two adjacent thin elastic films to calculate bending rigidity from the electromechanical response of the membrane to the applied field. Using DOPC, we show that bending rigidities determined by this approach are in good agreement with the existing work using neutron spin echo on vesicles, atomic force spectroscopy on supported lipid bilayers, and micropipette aspiration of giant unilamellar vesicles. We study the effect of asymmetric calcium concentration on symmetric DOPC and DOPG membranes and quantify the resulting changes in bending rigidity. This platform offers the ability to create planar bilayers of controlled lipid composition and aqueous ionic environment, with the ability to asymmetrically alter both. We aim to leverage this high degree of compositional and environmental control, along with the capacity to measure physical properties, in the study of various biological processes in the future.

## 1. Introduction

The cell membrane serves as a barrier to control the transport of molecules into and out of the cell. Phospholipids make up a significant portion of the membrane, forming a bilayer consisting of a hydrophobic core and a hydrophilic exterior. Membrane lipid content in a given cell varies both chemically and compositionally, the former referring to the chemical structure of a lipid molecule within the membrane, and the latter referring to the relative amount of such lipids contained within a bilayer. Excluding sterols, lipid chemical diversity arises from the variation in the polar head, the central group, and the hydrocarbon tails [1]. Each lipid species may contain a different distribution of charge within its headgroup, unsaturation within its acyl tails, and the molecular structure of each, which collectively determine the lipid intrinsic curvature, packing parameter, and phase transition temperature. Depending on the type of cell, the relative amounts of each lipid species in the membrane vary, with sometimes stark differences in the quantities of saturated lipids and/or charged lipids [1]. Collectively, the lipid composition of the cell membrane determines macroscopic membrane properties such as bending rigidity (*k_c_*) [2], compressibility [3] and stretching modulus [4]. Such properties govern a multitude of biophysical processes, including membrane protein function [5], vitamin B12 uptake [6], and drug efficacy [7]. For example, membrane elasticity may allow or prevent the attraction or repulsion between proteins due to membrane deformation, resulting in protein–protein interactions such as cooperative channel gating [8]. Thus, understanding the compositional effect of lipids on membrane properties is an important step in understanding these biological processes.

However, the elucidation of a cell membrane’s rigidity is a difficult task due to the vast domain space available for study, including but not limited to: lipid composition, temperature, ionic environment, membrane shape and asymmetry. Experimental and computational values of bending rigidities vary significantly, sometimes by orders of magnitude, across different methods [9]. In-depth comparisons of a wide assortment of membrane compositions under specific experimental conditions, such as aqueous ionic strength or temperature, are nearly impossible. The ability to obtain granular data within a given set of parameters is paramount to understanding and modifying membrane–membrane interactions. Experimental artificial lipid membranes provide an opportunity to selectively probe a set of parameters while excluding the additional complexity introduced by a complete cell membrane to begin revealing how macroscopic membrane properties correlate with biological function. 

As a result, there are various experimental methods to determine the bending rigidity of artificial lipid membranes: micropipette aspiration [10] or fluorescence fluctuation spectroscopy [11] of giant unilamellar vesicles (GUVs), scattering methods on lipid vesicle multilayers (neutron [12], X-ray [13]) or supported lipid multilayers (off-specular X-ray [14], neutron [15]), and atomic force microscopy (AFM) of supported lipid bilayers [16] (SLBs) or GUVs [17]. To determine bending rigidity using micropipette aspiration, GUVs are partially aspirated into a micropipette via a small pressure change, which results in a portion of length *L* entering the pipette. The increase in projected length is proportional to an increase in suction pressure and used to determine the apparent area strain, which in turn is used to calculate the bending rigidity. While micropipette aspiration externally deforms the membrane, fluorescence fluctuation, or flicker spectroscopy, measures the thermal undulations of the bilayer. Similarly, scattering approaches rely on measuring the natural bending fluctuations of membranes. Supported lipid bilayers are generally not free to undulate unless multilayers are created, but single supported bilayers are amenable to interrogation via AFM. The approach involves the adsorption of lipids onto a substrate to form a bilayer followed by the indentation of an AFM tip, with the slope of the resultant force curves corresponding to *k_c_*. This technique can be adapted to free-standing membranes by adhering in-tact vesicles to a substrate and performing similar analyses. Vesicles can be imaged before and after measurements take place to confirm the absence of probe damage. The indentation is carried out over a short distance (δ) and with little force (F), creating a force curve. The Young’s modulus ($E_{⟘}$) is extracted from the slope of the initial linear section of the force curve with the bending rigidity, then determined by modeling the vesicles as a thin shell with a Poisson ratio of 0.5 [18]. Despite the success of these methods, there is limited ability to integrate them with dynamic changes to the membrane environment, such as changes in the aqueous solution conditions, due to the involvement of a substrate or challenges accessing both sides of the bilayer independently.

Computational approaches to model membranes include using real-space fluctuations (RSF) [19] or field-theoretic umbrella sampling [20]. RSF is rooted in analysis of molecular dynamics (MD) simulations, but uses the fluctuations in the tilt and splay degrees of freedom sampled in MD trajectories to calculate thermodynamic tilt modulus and bending rigidity. The calculation of bending rigidity in the field theoretic umbrella coupling approach uses a non-equilibrium bent membrane’s spatially varying chemical potential and density after unbending and relates them to the free energy of a curved elastic surface. Both RSF and field-theoretic umbrella sampling (using the MARTINI model) yield values similar to those found in GUV systems. Field theoretic umbrella sampling using a coarse-grained amphiphile model, however, results in calculated rigidity values approximately four times lower.

We seek to determine the bending rigidity of large-area model biomembranes (LAMBs), which are planar, free-standing, membranes formed via a thin film balance technique. These are a variant of the common black lipid membrane (BLM) technique, with added stability and flexibility due to a microfluidic chip and pressure control system. Although bilayers formed through this method are amenable to measuring tension [21], introducing proteins [22], and probing phospholipid asymmetry [23], determining membrane bending rigidity has previously been a challenge and necessitates the development of a new approach. To accomplish this, we rely on the application of a voltage potential across the bilayer that results in membrane electrostriction and an increase in membrane area. By extending the approach of Alvarez [24] to accommodate changes in membrane volume, we use the voltage-dependent capacitance to calculate the membrane Young’s modulus. This direct measurement of the Young’s modulus is then used to deduce the membrane bending rigidity. We model the membrane as two flexible lipid sheets and use thin plate theory in the framework of linear elasticity to calculate their bending rigidity from the Young’s modulus and thickness. The bending rigidities calculated using this method agree with those found using the aforementioned experimental and theoretical approaches for DOPC bilayers. Determining the membrane bending rigidity for free-standing, planar membranes produced with this platform has a twofold importance. First, we can contribute to (and refine) existing literature values, which vary widely [9], and second, we can leverage the advantages of our system, in particular the independent control over the aqueous environment on either side of the membrane, to begin to understand how asymmetric ionic strength impacts the rigidity of symmetric zwitterionic and anionic membranes. Our initial results indicate that while zwitterionic DOPC membranes become less stiff upon the introduction of the asymmetric calcium concentration, anionic DOPG membranes stiffen as the calcium asymmetry increases.

## 2. Materials and Methods

### 2.1. Materials

1,2-dioleoyl-sn-glycero-3-phosphocholine (DOPC), and 1,2-dioleoyl-sn-glycero-3-phosphoglycerol (DOPG) in chloroform were obtained from Avanti Polar Lipids. Sodium Chloride (NaCl), calcium chloride, CaCl_2_, and sodium bicarbonate, NaHCO_3_, squalene and octadecyltrichlorosilane (OTS) were purchased from Fisher Scientific. Aqueous buffer consisting of 150 mM NaCl, 2 mM CaCl_2_, 0.2 mM NaHCO_3_ was prepared using ultra-pure water (Milli-Q, Millipore-Sigma, Burlington, MA, USA) and was filtered through a 200 nm pore filter before use.

### 2.2. Lipid Bilayer Preparation

We use a microfluidic device consisting of a stainless-steel capillary leading into a glass microfluidic chip. A single channel within the glass chip connects to the capillary tube, then bifurcates into two channels. Each channel then leads to a circular chamber with 24 channels, which lead to a 0.9 mm aperture. The chip must be functionalized to ensure proper pinning of the lipid-carrying oil at the aperture. The chip is immersed in 1 mM OTS in squalene overnight to hydrophobize the outside and inside surfaces. The device is then plasma cleaned briefly to partly remove the functionalization of the exterior surfaces, rendering them hydrophilic, while keeping the aperture hydrophobic.

Lipids (DOPC or DOPG) are prepared from stock dilutions in chloroform and dried in scintillation vials under nitrogen. The vials are subsequently placed under a vacuum (~5 mbar) overnight to remove the residual chloroform. Once dry, the lipids are resuspended in squalene to a final concentration of 2.5 mg/mL. This solution is then sonicated for at least 2–4 h to suspend and solubilize the lipid film, with an additional 2 h of sonication prior to the experiment.

The microfluidic chip is loaded with lipid–oil mixture and placed within a 3D-printed sample chamber. The capillary is connected to a microfluidic pump (ELVEFLOW Ob1 Mk3) and a pressure transducer (MKS 120AD) with readout (MKS PR4000B-F), enabling fine pressure control (±3 Pa). A thick oil film is first formed across the microfluidic device aperture. The film is flanked by a buffer on either side, creating two opposing monolayers of phospholipids. The thick oil film is thinned by the decreasing pressure, creating a phospholipid-laden thin film from which a bilayer nucleates. The bilayer is imaged with reflected 640 nm light (Lumencor Spectra X light engine) using a Nikon Ti2 Eclipse. A square-wave potential pulse is applied on either side of the membrane using electrodes attached to the headstage of a HEKA patch clamp amplifier. The square wave consists of 2 s on and 2 s off, starting at 1 mV and increasing by ±25 mV until the potential reaches ±200 mV. The applied potential increases both the membrane area and capacitance. Bilayer images and capacitance measurements are acquired simultaneously, allowing for the calculation of the hydrophobic membrane thickness at specified times from the corresponding capacitance and area.

### 2.3. Asymmetric Ion Composition Experiments

Typically, the artificial membrane is exposed to symmetric ionic conditions, consisting of 150 mM NaCl, 2 mM CaCl_2_, and 0.2 mM NaHCO_3_. Once the membrane forms, each monolayer separates the volume of buffer into top and bottom, allowing for selective tuning of buffer composition. We use this feature to explore the effects of ionic asymmetry by adding 50 µL of 200 mM CaCl_2_ to the top chamber. After each addition, we wait 5 min and compress the bilayer, as previously described. We repeat this process to further increase ionic asymmetry, while keeping the membrane intact.

## 3. Results and Discussion

LAMBs consist of two adjacent phospholipid monolayers with hydrophilic headgroups facing the buffer solution, and hydrophobic tails forming a dielectric core. This planar membrane can be modeled as a capacitor with capacitance, *C*:

(1) $$C=\varepsilon_{0}\varepsilon_{r\;}\frac{A}{d}$$

where $\varepsilon_{0}$ is the permittivity of free space, $\varepsilon_{r\;}$ is the dielectric constant (2.5), *A* is the area of the bilayer, and *d* is the thickness. With increasing voltage, increased compressive stress is applied to the membrane, resulting in a decreased thickness. The Young’s modulus, $E_{⟘}$, can therefore be calculated from the change in membrane thickness between its static value and its value under an applied field [25,26]:

(2) $$\Delta d=\frac{C_{m}\psi^{2}}{2E_{⟘}}$$

where Δd is the decrease in thickness due to an applied voltage, ψ, across the membrane, and $C_{m}$ is the specific capacitance of the membrane at 0 mV ($C_{m}=C_{0}/A\;$). This approach to determining the modulus yields consistent values for DOPC (~10^5^ Pa) [22,23] that are an order of magnitude lower than those reported in other systems [27] when hexadecane is used as the oil solvent. We suspect that this is partially due to the impact of residual oil molecules within the membrane, which causes the membrane thickness to swell [23,28,29,30], and also in part due to the membrane area (and therefore, volume) changing during compression. When squalene is used as the oil solvent, membrane thicknesses approach the solvent-free limit and measured Young’s moduli (~10^6^ Pa) are on the same order of magnitude as solvent-free approaches; however, the moduli values are still on the lower edge of the range, indicating possible remaining contributions from the membrane area (volume) changes during compression. We note that by keeping the oil solvent conditions constant and judiciously changing characteristics of the lipid bilayer or its external environment, the influence of residual oil can be negated and meaningful insights into membrane biophysics may still be accomplished.

An alternate approach to calculating the modulus presented by Alvarez [24] models the voltage-dependent capacitance of a bilayer, $C_{\psi }$, as:

(3) $$C_{\psi }=C_{0}\left[1+\alpha \psi^{2}\right]$$

where $C_{0}$ is the capacitance at ψ = 0 and α is a proportionality constant. However, in applying this model to a BLM, the inherent assumption of a constant membrane area was necessary despite an inability to simultaneously image the bilayer to confirm this assumption. Therefore, we adapt this model to our experimental system, which is able to measure changes in the membrane area in addition to capacitance. The increase in membrane area with voltage in the LAMB platform, or other oil solvent-based bilayer system, can be significant because lipid molecules can enter the membrane from the oil phase Plateau border, which stabilizes the membrane. This is in distinction to lipid bilayers with a constant number of molecules, such as vesicles, which are only able to accommodate relatively small (few %) changes in area before rupturing. Therefore, in order to apply Equation (3) appropriately, we must calculate the equivalent membrane capacitance considering only thickness (volume) changes when moving from ψ = 0 to another value. This is necessary because both the thickness decrease due to compressive stress and the membrane area increase contribute to the measured membrane capacitance via Equation (1).

We first define the volume of a lipid membrane, as its area times thickness, and rewrite Equation (1) as:

(4) $$C=\frac{\varepsilon_{0}\varepsilon_{r\;}V}{d^{2}}$$

The static capacitance and area measurements are used to calculate the initial bilayer thickness and membrane volume ($V_{0}$). As nonzero voltages are applied, we then measure the membrane thickness ($d_{\psi }$) and recalculate the equivalent membrane capacitance at a constant initial volume, as:

(5) $$C_{\psi }=\frac{\varepsilon_{0}\varepsilon_{r}\;V_{0}}{d_{\psi }^{2}}$$

Since the membrane area increases as the field is applied, Equation (5) adjusts the capacitance measurements to report only the contribution from the thickness change, and we refer to this value as the constant-volume capacitance. This is an important step in ensuring the modulus of the membrane is not being influenced by disproportionate growth in the area in response to the compression.

**Figure 1:**
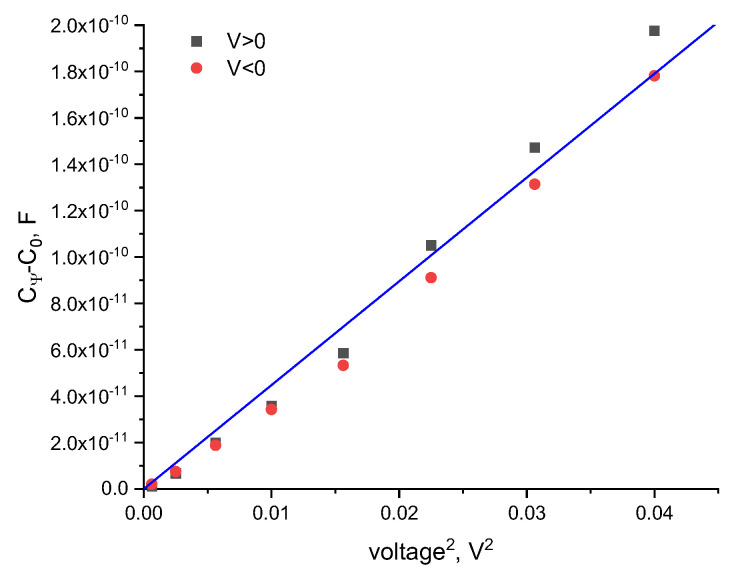
The voltage-dependent capacitance of a DOPC bilayer in hexadecane for both positive and negative applied voltages. The proportionality constant, α, is calculated by $\alpha =m/3C_{0}$.

Following Equation (3), a plot of the constant-volume capacitance against the square of the applied voltage results in a linear correlation, $\alpha =m/3C_{0}\;,$ where m is the slope. We determine α from the linear fit of the voltage-dependent volume vs. the voltage squared, as shown in Figure 1. By applying equal increasing positive and negative voltage it is apparent that, for symmetric bilayers in symmetric aqueous phase environments, the data overlays, as expected.

**Figure 2:**
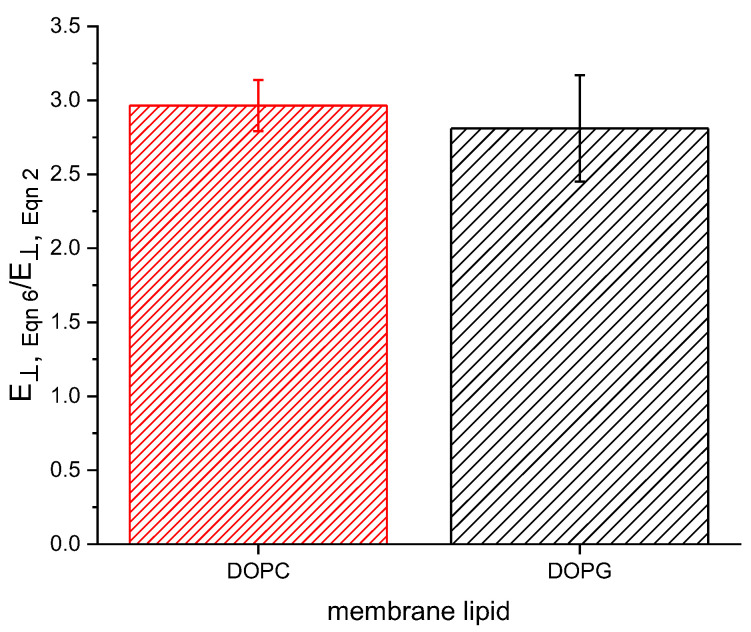
Young’s moduli calculated using Equation (6) are approximately three times larger than those calculated by Equation (2).

The proportionality constant can be used to determine Young’s modulus at a constant membrane volume, as described by White [31]:

(6) $$E_{⟘}=\frac{C_{m}}{\alpha d_{0}}$$

where $d_{0}$ is the thickness at ψ = 0. Comparing the Alvarez constant volume method to our previous [22] approach results in approximately a threefold increase in $E_{⟘}$, as shown in Figure 2. The compression resulting from an applied potential is responsible for both a decrease in thickness and an increase in area due to the new membrane being formed from lipids present on the Plateau border surrounding the initial bilayer. These two changes occur simultaneously and have previously been implicitly assumed to occur under constant membrane volume. However, we find that membrane area increases in a disproportionate manner when compared to the decrease in thickness, resulting in an increased volume with increasing field strength. This volume increase results in a lower calculated Young’s modulus following the method of Hianik (Equation (2)) [23].

To calculate the membrane rigidity from the measured Young’s modulus, we apply thin plate theory in the framework of linear elasticity [32] to planar phospholipid bilayers. For a membrane with Young Modulus, $E_{⟘}$, thickness d, and a Poisson ratio of *v*, the model gives

(7) $$K_{A}=\frac{E_{⟘}d}{\left(1-v\right)}$$

where $K_{A}$ is the area expansion modulus. The bending rigidity for the bilayer system is then

(8) $$k_{c}=\frac{K_{A}d^{2}}{24\left(1+v\right)}$$

**Figure 3:**
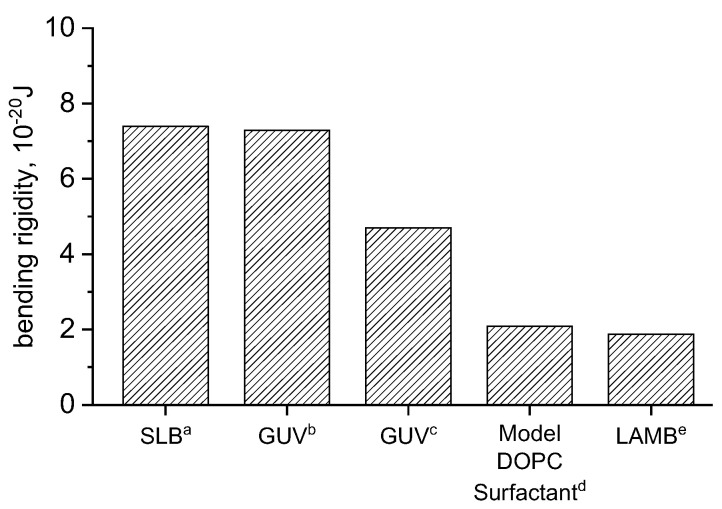
Comparison of bending rigidities of DOPC bilayers studied in different platforms: (a) ref [34], (b) ref [12], (c) ref [35], (d) ref [33], and (e) LAMB (this work).

The area expansion modulus and subsequently $k_{c}$ is calculated according to Equations (7) and (8) for DOPC in 150 mM NaCl, 2 mM CaCl_2_, and 0.2 mM NaHCO_3_, and compared to similar experimental and simulations results (Figure 3). The result is in good agreement with simulations and slightly lower than experimental results. A study combining measurements on entropically stabilized DOPC vesicles in NaBr and self-consistent field theory [33] determined bending rigidities of 2.09 × 10^−20^ J. De Mel studied [12] DOPC vesicles using neutron spin echo (NSE), finding rigidities of 7.29 × 10^−20^ J and 13 × 10^−20^ J for DOPC vesicles in water and 150 mM NaCl, buffer, respectively. Studies carried out by Picas [34] in SLBs produced similar results to those of De Mel [12]. Other [35] GUV studies found values of k_c_ ranging from 4–5.3 × 10^−20^ J. Our large area biomembrane (LAMB) experiments were performed in a buffer consisting of 150 mM NaCl, 2 mM CaCl_2_ and 0.2 mM NaHCO_3_. Its bending rigidity is in good agreement with values determined by Claessens [33]. 

Values found in literature contain a wide spread, with some studies of GUVs using fluctuation analysis [36] reporting an even higher bending rigidity of 11 × 10^−20^ J for pure DOPC vesicles. Computational approaches yield a bending rigidity of 7.5 × 10^−20^ J (18.3 k_B_T) for DOPC membranes using RSF [19], and 8.3 × 10^−20^ J using field theoretic umbrella sampling [20]. Small vesicle fluctuation/deformation studies [37] reported a statistically significant *k_c_* dependence on vesicle size, measurement method, sample cell set-up, and temperature. Bending rigidities of DOPC vesicles ranged from 1.6 ± 0.13 × 10^−20^ J to 4.4 ± 0.44 × 10^−20^ J, with a fluctuation mode analysis and 6.1 ± 0.85 × 10^−20^ J to 8.2 ± 0.90 × 10^−20^ J when using the deformation analysis. Values calculated using fluctuations align with our results, while those calculated using deformation are close to refs [12,34]. 

The challenge in obtaining direct comparisons for the membrane bending rigidity of even simple monocomponent bilayers such as DOPC arises in part due to variations in the aqueous buffer environment necessitated by the different experimental platforms. Ionic strength is known to alter the bending rigidity of vesicles [38,39] and their size [40]. Vesicle bending rigidity reportedly decreases as the NaCl concentration increases from 0 mM to <25 mM [39], with the trend plateauing at above 100 mM [12]. Further complications arise from the diversity of phospholipids, which can differ in the headgroup charge, tail length, saturation, and isomerism. The totality of these parameters results in broad differences of the reported *k_c_*. Our reported values are in line with those modeled by Claessens [33] and Niggemann [37] although the salt and its concentration vary. 

**Figure 4:**
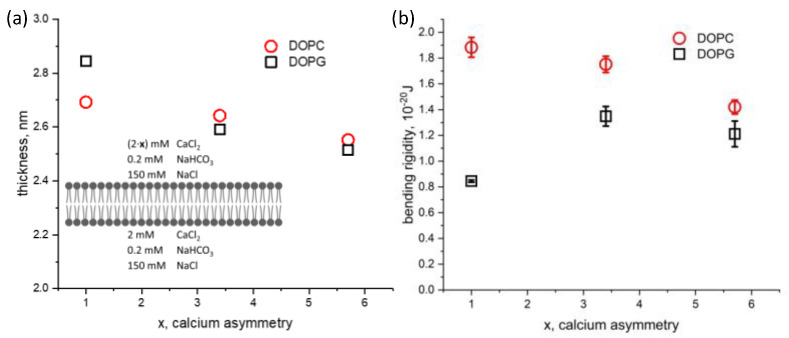
Dependence of (**a**) hydrophobic membrane thickness and (**b**) membrane bending rigidity on the degree of calcium asymmetry factor, *x*, in the aqueous phase surrounding symmetric DOPC and DOPG bilayers. The factor *x* represents the degree of calcium asymmetry across the membrane, ranging from 2 mM/2 mM (*x* = 1, symmetric) to 10.4 mM/2 mM (*x* = 5.7, asymmetric), as shown in the inset schematic.

Next, the effect of the ionic solution asymmetry on membrane thickness and bending rigidity was studied for both DOPC and DOPG bilayers. Calcium chloride was added into the top chamber to increase its concentration from 2 mM to 11.4 mM, while keeping the solution conditions constant on the opposite side of the membrane ([Ca^2+^] = 2 mM). We define the calcium ionic strength asymmetry factor, *x*, which is the ratio between the calcium concentration on the top and bottom aqueous chambers. This factor ranges from 1 for symmetric conditions to nearly 6 in our experiments. The hydrophobic thicknesses of DOPC (2.69 nm)and DOPG (2.84 nm) bilayers in symmetric solution conditions agree with our previous results using squalene [22] and approaches the solvent-free limit measured by others [41,42] (Figure 4a). Asymmetric calcium conditions result in a similar thinning of the membrane for both DOPC and DOPG. In contrast, we find the effect of asymmetric calcium concentration to have different effects on the bending rigidity depending on the lipid charge (Figure 4b). The bending rigidity of DOPC decreases approximately 25% from 1.9 × 10^−20^ J to 1.4 × 10^−20^ J. The bending rigidity of DOPG under symmetric aqueous conditions agrees well with off-specular neutron scattering of multilayer stacks [43], albeit with different ionic and hydration conditions. Bolik’s stacked bilayers exist at an air interface, with 100% relative humidity, while ours are surrounded by aqueous buffer. Upon introducing asymmetry in calcium concentration, the bending rigidity of DOPG increased by 50%, from 0.8 × 10^−20^ J to 1.2 × 10^−20^.

Calcium is known to associate with both zwitterionic and anionic membranes; weakly with the former, and strongly with the latter [44]. Monovalent cations been reported to “soften” zwitterionic POPC GUVs grown in sucrose and later exposed to external NaCl and KCl [45]. The effect is attributed to a reduction in the effective molecular area due to ions inciting a local condensation of lipids. When the asymmetric calcium concentration increases within our DOPG LAMBs, we observe a stiffening of the membrane. This behavior has been noted in DOPG GUVs and is attributed to the interplay between electrostatic leaflet repulsion and lipid area packing [40]. The *k_c_* of an anionic, salt-free bilayer is expected to decrease with the initial addition of salt, resulting from a dehydration of the bilayer accompanied by a reduction in the electric double layer. The subsequent addition of salt is thought to increase the bending rigidity due to a reduction in the lateral mobility of DOPG that includes an area-driven stress in addition to the dehydration effect dominating any further decreases in the electrostatic repulsion [46,47]. The area-driven stress in DLPC bilayers described by Deserno [47] arises from asymmetry in the number of lipids within each monolayer, resulting in increased *k_c_*. Similarly, an increase in calcium concentration on one side of the membrane may lead to stiffening from the lowered DOPG lipid area, causing asymmetry in the number of lipids per monolayer.

## 4. Conclusions

The bending rigidity of DOPC lipid bilayers formed in a planar, freestanding, membrane platform has successfully been determined from Young’s modulus electrostriction data and found to be in good agreement with existing experimental values. The application of an electric stress on a planar membrane causes both areal and thickness changes, which must be taken into account in order to accurately determine the Young’s modulus and bending rigidity from capacitance measurements. Using this approach, the behavior of DOPC and DOPG membranes with asymmetric CaCl_2_ conditions was investigated, demonstrating opposite effects. This is rationalized based on the specific interaction of calcium ions with anionic and zwitterionic phospholipids, which can either cause a reduction in lateral lipid mobility or local mean molecular area, respectively. We expect to utilize this method for additional bending rigidity characterization of more complex asymmetric membranes (lipid; ion) and alternate membrane compositions, including different tail lengths, saturation and headgroups, in the future. By characterizing membrane physical properties simultaneously with controlling the lipid membrane environment, this will aid in future work related to linking the change in physical properties with biological function.

## Data Availability

The raw data used in this study is available upon request.
